# Applications of Graphene and Its Derivatives in the Upstream Oil and Gas Industry: A Systematic Review

**DOI:** 10.3390/nano10061013

**Published:** 2020-05-26

**Authors:** Lipei Fu, Kaili Liao, Bo Tang, Lujun Jiang, Weiqiu Huang

**Affiliations:** School of Petroleum Engineering, ChangZhou University, Changzhou 213164, China; fulipeiupc@163.com (L.F.); tangbo@cczu.edu.cn (B.T.); jlj18351205521@163.com (L.J.)

**Keywords:** graphene and its derivatives, upstream oil and gas industry, enhanced oil recovery, well working fluid, profile control and water shutoff, oily wastewater treatment

## Abstract

Graphene and its derivatives, with their unique two-dimensional structures and excellent physical and chemical properties, have been an international research hotspot both in the research community and industry. However, in application-oriented research in the oil and gas industry they have only drawn attention in the past several years. Their excellent optical, electrical, thermal and mechanical performance make them great candidates for use in oil and gas exploration, drilling, production, and transportation. Combined with the actual requirements for well working fluids, chemical enhanced oil recovery, heavy oil recovery, profile control and water shutoff, tracers, oily wastewater treatment, pipeline corrosion prevention treatment, and tools and apparatus, etc., this paper introduces the behavior in water and toxicity to organisms of graphene and its derivatives in detail, and comprehensively reviews the research progress of graphene materials in the upstream oil and gas industry. Based on this, suggestions were put forward for the future research. This work is useful to the in-depth mechanism research and application scope broadening research in the upstream oil and gas industry.

## 1. Introduction

With the continuous growth of population and the rapid development of economy, the global energy demand is increasing. Oil and natural gas, as the most important current energy sources for human society, will not be replaced on a large scale, at least during the first 50 years of the 21st century. The oil and gas industry is a multibillion-dollar market that affects the lives of anyone who has ever driven a car, used electricity, walked on asphalt-paved roads or used polymer products such as plastics [1]. Therefore, new technologies need to be actively explored to increase the production of fossil fuels and meet the needs of rapid economic growth and human life.

In recent years, nanomaterials application research in biology, medicine, electronic engineering and other fields has made rapid progress, which also provides new ideas for the development of the upstream oil and gas industry. The relatively high cost of nanomaterials is a major challenge for their application in the oil and gas industry, but scholars and oil companies around the world are still actively carrying out research on the applications of nanomaterials in the oil industry. The most widely studied nanomaterials are carbon nanotubes, carbon nanofibers, nano-oxides, and nano-clays [2].

As a special nanomaterial, the application of graphene and its derivatives in the field of oil and gas industry has attracted more attention. However, compared with its extensive application in other fields (such as biodegradation, biomedicine, energy storage and conversion electronic devices, electronics, photonics, high-strength composite materials, etc.), the application of graphene in oil and gas exploration and production is still at the initial stage. Although its research time is short, the effects of graphene and its derivatives in oil exploration and development are obvious, so many scholars and engineers believe that they will have a better application prospects in the upstream oil and gas industry. Although in theory, graphene should be large in size and large in area, each carbon atom is sp^2^ hybridized, and there are dangling bonds on the edges. However, because graphene needs to be stable in the natural environment, the hanging bonds on the edges of such nanoscale graphene sheets are occupied by other chemical bonds, therefore, in practical applications, it is always a three-dimensional graphene material composed of nanoscale graphene sheets. In this paper, the application of graphene and its derivatives in petroleum engineering is reviewed, and their development directions in the upstream oil and gas industry are proposed.

## 2. Characteristics of Graphene Materials

### 2.1. Graphene and its Derivatives

In 2004, Novoselov and Geim at the University of Manchester in the UK obtained the first graphene sheet by a mechanical exfoliation method, and since then graphene has become a research hotspot in the scientific community around the world [3]. The unique properties of graphene and its derivatives make it of great research and application value. Graphene is composed of a single layer of carbon atoms linked by sp^2^ hybridization. Its basic structural unit is the most stable benzene-like six-membered ring structure. Its theoretical thickness is only 0.35 nm, which is the thinnest two-dimensional material discovered so far [4].

Graphene has many excellent physical and chemical properties. For example, the strength of graphene is the highest among the tested materials, reaching 130 GPa, which is more than 100 times that of steel. Its carrier mobility is 1.5 × 10^4^ cm^2^·V^−1^·s^−1^, which is twice that of indium antimonide material (the material that has the highest carrier mobility known so far) and 10 times higher than that of the commercial silicon wafers. Under certain conditions (such as low temperature quenching, etc.), the mobility can even be as high as 2.5 × 10^5^ cm^2^·V^−1^·s^−1^. The thermal conductivity of graphene can reach 5 × 10^3^ W·m^−1^·K^−1^, which is three times that of diamond. In addition, graphene has excellent chemical stability and adsorption properties [5,6,7,8,9].

Graphene derivatives generally include graphene oxide (GO), reduced graphene oxide (rGO), graphene quantum dots (CQDs), doped graphene and modified graphene, among which graphene oxide is the most widely used graphene derivative. Graphene oxide is a kind of single-layer two-dimensional material, obtained by the oxidizing and intercalating modification treatment of graphene, which changes part of carbon atoms from sp^2^ hybrid state to sp^3^ hybrid state, and then peeled layer by layer [10]. The structure of graphene oxide is similar to that of graphene, carrying a large number of active oxygen-containing functional groups, such as hydroxyl, carboxyl, epoxy, etc., which enables GO to not only have the characteristics of graphene, but also have other chemical and physical properties (for example, graphene oxide can be evenly and stably dispersed in water). At the same time, due to the introduction of oxygen-containing functional groups, the large π conjugate structure of graphene is destroyed, so the ability of conducting electrons is lost, and the conductivity is obviously reduced. Most of the oxygen-containing groups in reduced graphene oxide were removed, and the C/O ratio increased significantly, therefore, it turns from being hydrophilic to hydrophobic [11,12,13,14].

### 2.2. Behavior in Water

The industrial applications of graphene and its derivatives are mainly in the fields of surface coating and preparation of nanocomposites, in which graphene and its derivatives participate the whole process mainly in the dispersion form. The research on the behavior of graphene and its derivatives in water is involved in the fields of chemistry, materials science, environmental science and biomedicine [15,16]. In the field of oil and gas production, in order to be applied to well working fluids and chemical enhanced oil recovery, graphene and its derivatives in the form of nanoparticles are usually dispersed in a water phase. Therefore, their behavior in water is particularly important for their application in the oil and gas industry.

The solubility of graphene in water is very low. Due to the effect of van der Waals forces, it easily forms agglomerates or even accumulates to form turbostratic carbon in aqueous solution [17]. The zeta potential on the surface of graphene is affected by the pH value of the solution, and the interaction between the particles is also affected, so the dispersion state of graphene in water can be changed. However, GO particles are easy to disperse evenly in aqueous solution, due to their hydrophilic oxygen-containing functional groups on the surface [18]. In addition, GO tends to form stable colloidal solutions in water on account of the electrostatic stabilization mechanism. Lanphere et al. showed that when the pH value was 5–9, the pH value had no significant effect on both the hydraulic diameter and the average electrophoretic mobility of GO. However, when the pH value reduced from 4 to 2, the average electrophoretic mobility of GO was significantly reduced, the electrostatic repulsion on the surface was weakened, and the hydraulic diameter of GO was sharply increased [19]. In addition, the average electrophoretic mobility and hydraulic diameter of GO increased with the increase of ionic strength. When the ionic strength of the solution was greater than 0.1 mol·L^−1^, GO began to coagulate rapidly. Furthermore, the influence of divalent metal ions on the average electrophoretic mobility and the hydraulic diameter was stronger than that of univalent metal ions. Moreover, the humic acid had a significant effect on the stability of GO in the solution. When the concentration of humic acid increased from 0 to 10 mg·L^−1^, the hydraulic diameter of GO decreased from 500 nm to 250 nm, and the stability was significantly improved [20]. In addition, recent study conducted by Szabo et al. reported that the limit of rapid aggregation was found to be size and composition dependent. They also pointed out that not only the widely employed Hummers-Offeman method, but the overlooked Brodie method is also capable of providing graphene oxides with good dispersion stability in water [21].

### 2.3. Toxicity in Organisms

With the rapid growth of the application scope and usage of graphene materials, it is inevitable they will leak into the environment at some point during the whole cycle of production, storage, transportation, use, disposal and recycling. Although graphene nanomaterials have relatively good biocompatibility, they still have certain biological toxicity. Therefore, the impact of graphene nanomaterials on organisms and the environment has attracted more and more attention [22]. The oil and gas industry itself has the characteristics of high pollution and high risk. Therefore, when graphene and its derivatives are used in the oil and gas industry, their biological toxicity must be considered, thus ensuring that the oilfield enterprises always stick to the people-oriented principle and develop products towards the direction of environmental protection and safety. Current biological toxicity studies on graphene and its derivatives mainly include their cytotoxicity, animal toxicity, plant toxicity and antibacterial properties, etc. [23,24]. Clarifying these charcateristics is of great significance for the functionalization and practical application of graphene.

Cytotoxicity is one of the important indexes used to evaluate the safety of pollutants. Studies have shown that graphene nanomaterials have certain cytotoxicity, and their toxicities are closely related to their physical and chemical properties and the types of cells, and it also has a significant concentration dependence. The mechanism of the cytotoxicity of graphene is not very clear at present, but studies have shown that oxidative stress and inflammatory reaction are the main mechanisms leading to cell death. Graphene materials have high surface activity, which can produce a lot of reactive oxygen radicals (ROS) after aggregation on the surface or inside of cells. These ROS further lead to DNA fragmentation, cell membrane damage and mitochondrial dysfunction [25]. Qu et al. also found that GO can increase ROS levels in cells when it enters cells and accumulates to a certain extent. At the same time, go can also interact with the toll-like receptor 4 (TLR4) on the surface of cell membrane and activate TLR4 signaling pathway, which leads to inflammation and programmed cell death. Qu et al. also found that after GO entered the cell and accumulated to a certain degree, it could increase intracellular ROS. At the same time, GO also interacted with the toll-like receptor 4 (TLR4) on the cell membrane surface, and activate the TLR4 signaling pathway, thereby triggering an inflammatory response and leading to programmed cell death. In addition, GO can directly damage the cytoskeleton, change the cell morphology and affect the normal function of cells [26]. Wang et al. found that the toxicity of GO aqueous solution was not obvious at a low concentration of 20 μg/mL, but increased significantly when the concentration was more than 50 μg/mL [27].

Graphene materials have also shown some toxic effects on animals. At present, most of the studies focus on mammals such as rats and mice. The lower protozoa and nematodes, as well as zebrafish and other aquatic animals have also been studied. The toxicity of graphene materials to animals is closely related to its action position, action mode and action concentration, as well as the size of itself and the types of surface functional groups. The toxicity of graphene to mammal is manifested as low acute toxicity. GO is more toxic to the lungs of mammals than graphene, however, the surface modification can avoid the toxic effects of GO. The size of graphene material is also a major factor affecting its toxicity [28,29,30,31]. In addition to mammals, Zhao et al. found that GO had obvious reproductive toxicity to the nematode *Caenorhabditis elegans*, which can damage its gonad development [32]. Chen et al. studied the effect of GO on zebrafish and the results showed that GO had no obvious acute toxicity to zebrafish but could lead to oxidative stress and immune toxicity in this species [33].

Graphene materials are toxic to both terrestrial plants and algae. Begum et al. studied the toxic effects of graphene on a variety of vegetables, from the aspects of growth, biomass, shape, cell death and active oxygen level of their roots and buds. The results showed that higher concentrations of graphene could significantly inhibit plant growth and biomass increase. In addition, different kinds of plants and different parts of plants have different reactions after exposure to the action of graphene [34]. Zhao studied the toxicity and translocation of GO to *Arabidopsis thaliana*, and found that GO had no significant effect on seed germination, seed development, seedling root development and flowering time. The above studies indicate that the toxic effect of graphene on plants may be related to oxidative stress, but the detailed mechanisms need further study [35]. Nogueira et al. studied the effect of GO on the green algae *Raphidocelis subcapitata* from the aspects of ROS, membrane damage and chlorophyll content, and the results showed that GO could lead to the increase of ROS and membrane damage in green algae cells, and the accumulation of graphene would also affect the lighting efficiency of green algae [36]. Ouyang et al. studied the toxic effect of GO on *Chlorella vulgaris* and found that GO could accumulate on the surface of algal cells, significantly reduce the permeability of cell membrane, and cause a degree of extent oxidative stress, resulting in algal cells damage [37]. The above studies have shown that the toxicity of graphene materials to algae is mainly caused by the increase of reactive oxygen species. In addition, the shadowing effect of graphene accumulation also affects the growth of algae.

In the aspect of antibacterial activity, Hu et al. first reported the antibacterial activity of GO and rGO, and found that the graphene-based nanomaterials can effectively inhibit the growth of *Escherichia coli* while showing less cytotoxicity. Graphene paper sheet obtained by simple vacuum filtration using graphene suspension also had good antibacterial effect [38].

In conclusion, the toxicity of graphene materials to cells, animals, and plants is relatively low, and they can show good biocompatibility after surface modification. Graphene has a good antibacterial effect on microorganisms. However, Schinwald et al. found that GO could interact with proteins in the plasma to form GO-protein composite particles. Graphene sheets could easily become inhalable particles, enter the lungs and deposit into the alveoli, and produce toxic effects on the lungs, causing severe pulmonary fibrosis and cysts. In addition, the particles may accumulate in the lung, liver, spleen and kidney. What makes matters worse is that it cannot be removed from the kidney [39]. Therefore, special care should be taken when handling graphene and its derivatives, and gloves, laboratory suits and protective masks should be worn.

## 3. Application in the Upstream Oil and Gas Industry

Exploratory research for applying graphene and its derivatives in the fields of well working fluids, chemical enhanced oil recovery, heavy oil recovery, profile control and water shutoff, tracer, and oily wastewater treatment, etc., was conducted in the past three years. Meanwhile, they have also been applied in tools, sensors and pipelines. Although the above research is still in the initial stages, we have reasons to believe that graphene and its derivatives will certainly provide new breakthroughs in their application in the oil and gas industry, because of their widely application research in many fields such as biomedicine, nanotechnology, electronics, photonics, heat transfer and so on, through which the similar mechanisms could be found.

### 3.1. Well Working Fluid

#### 3.1.1. Drilling Fluid

Drilling engineering is an important step in establishing a connection channel between the ground production system and the underground reservoir, which refers to the rock stratum with pores, fractures or holes, where oil or natural gas are stored and flow. The performance of the working fluid directly affects the construction work. Drilling fluid in the broad sense is all fluids (such as liquid, gas, foam) that help to produce and remove cuttings from the wellbore during drilling process. The functions of drilling fluid mainly include flushing the bottom of the well, carrying cuttings, balancing formation pressure, cooling and lubricating the drill bit, stabilizing the well wall, protecting oil and gas reservoir, and transmitting the ground power, etc. Monteiro et al. pointed out that graphene particles can effectively improve the fluid fluidity, filtration, rheology, stability, lubricity, electrical properties, thermal properties, viscosity and other properties, therefore it can be added to drilling fluid, completion fluid, cementing fluid, etc. [40]. This section focuses on the role of graphene and its derivatives in improving the filtration loss property, lubricity, and rheological property, and the borehole wall stability.

##### Filtration Loss Properties

Drilling fluids can be filtered into the reservoir at a certain temperature and pressure difference. The filtration loss property of a drilling fluid refers to the property of whether a drilling fluid can be easily filtered into the formation or not. A large amount of filtrate invading into the reservoir will block the oil/gas flow channels and cause damage to the reservoir. At the same time, the thick filter cake (drilling fluid filter cake is the solid phase deposited on the formation surface during the filtration process of the drilling fluid, which is measured by thickness, and also known as mud cake) may cause drilling accidents such as undergauging of holes, increased friction, bit freezing, and well leakage. Therefore, it is necessary to add a fluid loss control agent to drilling fluids to ensure the formation of high-quality mud cake and prevent the filtrate from invading the formation during the drilling process [41].

Submicrometer-thick membranes made from graphene oxide can be completely impermeable to liquids, vapors, and gases, including helium, but these membranes allow unimpeded permeation of water [42]. If it can be dispersed in the drilling fluid at nanometer scale, its huge specific surface area will make it adhere to the surface of the well wall at a very low concentration, and form a thin and tough integrated film through the connection way similar to tiles [43,44]. The film plays the role of sealing the micro-nanoscale pores in the formation and can be easily removed by backflow during production [45,46]. Therefore, graphene can be a powerful filtration loss control agent.

Graphene oxide, as a kind of nanomaterial with the same lamellar structure as graphene, is more suitable for use in water-based drilling fluid than graphene because its surface is rich in polar groups and it has good water dispersibility [47]. During the drilling, the filtrate of the drilling fluid penetrating into the pores of the formation begins from the moment that the drill bit breaks the formation to form a wellbore. Meanwhile, the solid particles adhere to the well wall to form a filter cake. The thickness of the filter cake is related to the amount of fluid loss. In general, the greater the filtration loss, the thicker the filter cake. Adding GO into the drilling fluid can improve its filtration loss property and improve the quality of the filter cake. Jamrozik et al. used scanning electron microscope (SEM) to compare the microstructure of filter cakes before and after adding 1.5 wt % GO to a low solid drilling fluid. The results showed that due to the existence of large number of oxygen-containing functional groups in GO, it easily reacted with the polymers in the drilling fluid, which made the surface of carbonate mineral microcrystalline in the filter cake become covered with a coating of GO-modified, highly polymerized polymers. Therefore, the formed filter cake was more compact, which could prevent water from entering the reservoir and was more conducive to stabilizing the well wall [48].

Kosynkin et al. believed that even if GO was added to a water-based drilling fluid at a dosage as low as 0.2 wt %, it still showed good filtration loss control performance. American Petroleum Institute (API) filtration loss is a commonly used parameter to describe the filtration performance of drilling fluids. API filtrate loss refers to the filtration loss of drilling fluid permeated through the filter paper with the cross-sectional area of 45.8 cm^2^ and the diameter of 9 cm at room temperature and 0.689 MPa pressure for 30 min. Compared with standard bentonite (API filtration loss was 7.2 mL, filter cake thickness was 280 μm), the best filtration loss property was achieved when the mass ratio of flaky GO to powdered GO was 3:1, and the results were that the API filtration loss was 6.1 mL and the filter cake thickness was 20 μm [49]. Xuan et al. pointed out that nano-GO could improve the filtration loss effect by increasing the viscosity and exhibited a stronger filtration loss control performance even at a lower concentration. Compared with the traditional filtration loss control agent, it was found that if nano-GO was used as the filtration loss control agent without the addition of bentonite, the API filtrate loss was reduced from 137.0 mL to 14.7 mL when the dosage of nano-GO was increased from 0.2% to 0.6%, which were obtained based on the experiments conducted according to the API standard procedure recommended for testing drilling fluids. Therefore, it was considered that nano-GO had excellent filtration loss control effect without needing the presence of bentonite [50].

##### Lubricity

Graphene has excellent self-lubricating properties, so it can be used to improve the lubricity of drilling fluids. Zhao et al. studied the lubrication and wear properties of graphene particles as lubricant additives added into the PAO4 lubricating oil by means of a UTM-2 tribometer via ball on plate contact reciprocating sliding. The results showed that the friction coefficient and the wear rate can be reduced by 78% and 95%, respectively, when graphene was added [51].

Taha et al. developed nanographene particles that can enter the pores of metals where they crystallize under high pressure to form a protective film, and prevent the formation of balled bits. The tests showed that the extreme pressure lubrication coefficient of water-based polymer drilling fluid could be reduced by 80% after adding graphene with a volume fraction of 1–5%, while it as only reduced by 30–40% after the addition of a common ester-based lubricant. In addition, graphene also can significantly improve the reservoir protection performance of the well completion fluid. After the interaction of graphene-based completion fluid with the reservoir, the reservoir permeability recovery rate reached 41%, while it was only 5% for a conventional completion fluid. After adding 2% graphene into the drilling fluid of a pilot well in Myanmar, the penetration rate increased from 3.0–4.0 to 9.0 m/h, while the friction resistance decreased by 70–80%. Meanwhile, the bit life was extended by 75%, the bit wear was small, and no mud was found on the bit surface. The wear rate of the drill bit was reduced, and no balled bit were seen [52,53].

Furthermore, Liu et al. pointed out that GO had a better effect on improving the lubricating properties and friction reduction of drilling fluid than graphene. A field application test in Northeast China showed that when 0.075 wt % GO was added to an oil-based drilling fluid, the lubrication factor of the drilling fluid was reduced by 15.6%, the reduction rate of the friction coefficient was increased by 24.3%, and the aluminum disk wear was reduced by 20.5% [54].

##### Rheological Properties

Halliday et al. pointed out in their patent that adding graphene particles to the drilling fluid could improve the rheological properties of drilling fluids, such as plastic viscosity, yield pint, gel strength, apparent viscosity, and so on. Using graphene particles 80 mesh size (the particle size distribution was greater than 65%) they could prepare a drilling fluid system with excellent rheological properties, while particles of 120 mesh could be used to prepare a stuck-freeing spotting fluid [55].

##### Borehole Wall Stability

Borehole wall stability refers to the ability of the face of a well to maintain its original state. Shale inhibitors are often used to inhibit shale expansion and maintain borehole wall stability. Graphene and its derivatives can also improve the shale stability [56,57,58]. Aftab et al. comparatively studied the expansibility of shale samples treated in five drilling fluid samples with KCl, KCl/partial hydrolytic polyacrylamide, KCl/graphene platelet, KCl/nanosilica, and KCl/multi-walled carbon nanotubes as shale inhibitors, respectively. X-ray diffraction results showed that the volume expansion rate of shale samples which were immersed in the drilling fluid with KCl/graphene nanosheets for 20 h was minimal, indicating that the addition of graphene to a drilling fluid could improve the shale stability [59]. Rana et al. studied the surfactant-modified GO to improve the performance of water-based drilling fluids. They claimed that the mechanism was that the sodium dodecylsulfate-modified GO (SDS-GO) could be adsorbed on the clay surface by electrostatic action, which could reduce the repulsions between clay particles. Compared with the conventional drilling fluids, the rheological properties and anti-swelling properties of SDS-GO-containing water-based drilling fluids were all improved [60].

#### 3.1.2. Cementing Fluid

**Figure 1:**
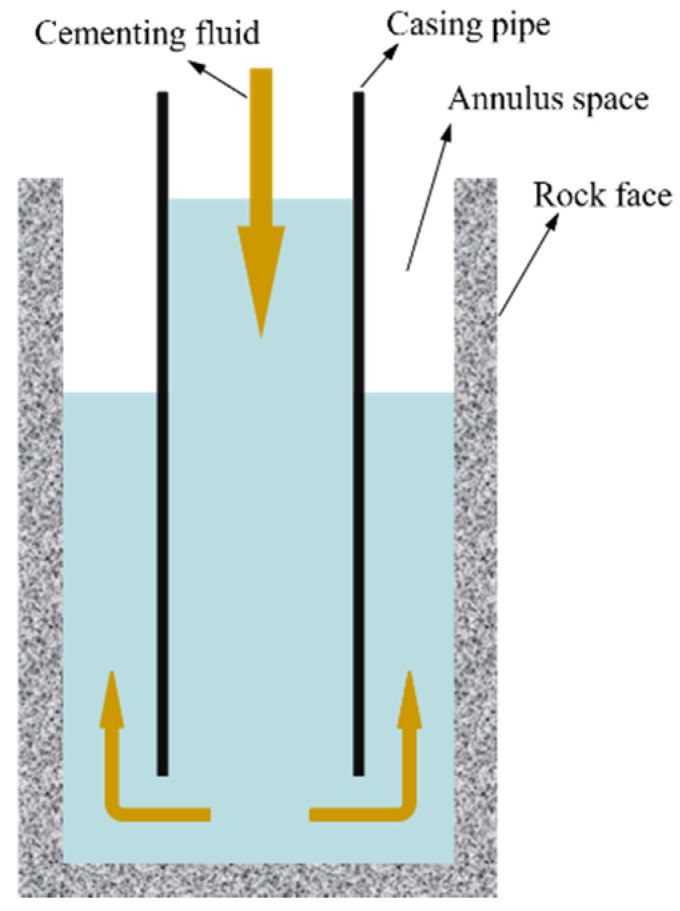
Schematic of cementing fluid flows in the annulus space.

Cementing fluid is the working fluid used in well cementation. During the process of cementing, the cementing fluid is injected from the casing pipe to the annulus space (as shown in Figure 1) between the rock face and the casing pipe, returns to a certain height, and then becomes a cement paste to consolidate the well wall and the casing. The main components of the cementing fluid include water, cement and other additives [61,62]. Graphene can improve the rheology of cementing fluids. Wang et al. conducted a quantitative study of the influence of GO dosage on rheological parameters of the cementing fluid by using rheometer and laser scanning confocal microscope. The results showed that the dispersed cement particles agglomerated again and formed a recombination flocculation structure under the influence of GO, which will affect the rheological properties of the cementing fluid; the thixotropy, plastic viscosity and yield stress of the cementing fluid were significantly increased after the addition of GO, therefore the stability of the cementing fluid was also greatly improved [63].

The strength of the cement sheath is the main criterion to evaluate the quality of cementing [64,65,66]. It is found that GO sheets had good hydrophilicity and can be dispersed in the cementing fluid as a reinforcing material, which is helpful to improve the bonding between cement hydration products, and prevent the initiation of microcracks and further growth in the early stage of cracking [67]. The mechanical properties of Portland cement can be improved (compressive strength and tensile strength increased by 10% and 30%, respectively) by changing the internal microstructure of cement after hydration. In addition, graphene’s high thermal conductivity and high electrical properties can reduce the chemical shrinkage of cement, which is conducive to controlling the growth of microcracks in the cement matrix and improving wellbore integrity [68,69].

#### 3.1.3. Fracturing Fluids

Unconventional oil and gas reservoirs, such as tight gas, tight oil, shale gas and shale oil, are hot topics in global energy development. The typical characteristic of such reservoirs is that the permeability is very low, so some reservoir stimulation measures are needed for hydrocarbon production. Hydraulic fracturing technology is the most important production stimulation treatment for the development of unconventional oil and gas reservoirs [70,71,72,73]. A fracturing fluid is the working fluid used in fracturing stimulation, and its performance directly affects the effect of fracturing stimulation. In order to improve the fracturing effect of unconventional reservoirs, scholars have done a lot of optimization research on the formulae of fracturing fluids. With the application of nanotechnology in the oil and gas industry, graphene has been applied to fracturing fluids by oil companies [74,75,76].

Saudi Arabian Oil Company (Dhahran, Saudi Arabia) pointed out in their patent that both nanographene and nano-GO can be used as crosslinkers for fracturing fluids. When a composite crosslinker system based on the above materials and crosslinkers was used to conduct fracturing, the shear resistance of fracturing fluids was improved by the synergistic effect between nano-GO or nanographene and cross-linking agents [77]. Lv et al. studied a GO-stabilized ultra-dry CO_2_ foam fracturing fluid, and the results showed that the addition of GO improved the thermal stability and effective viscosity of the ultra-dry CO_2_ foam. When GO and saponin were mixed, the interfacial viscoelastic modulus of CO_2_/liquid increased, and the interfacial bubble membrane appeared as a solid phase. Compared with pure surfactant-stabilized foam, GO improved the filtration loss of ultra-dry CO_2_ foam. When the filtration pressure difference was 3.5 MPa, the addition of GO significantly reduced the filtration loss coefficient of ultra-dry CO_2_ foam. The filter cake would be formed after the filtration loss of GO-stabilized CO_2_ foam, and the damage rate to the core was higher than that of the pure surfactant stabilized foam. However, the permeability damage rate of the core with permeability of 1.83 × 10^−3^ μm^2^ was still less than 10%, which was also clean for the reservoir. This research provided a new efficient fracturing system for unconventional oil and gas in water-deficient areas [78]. The above studies indicate that graphene materials can improve the performance of fracturing fluids.

### 3.2. Chemical Enhanced Oil/Gas Recovery

Fossil energy sources such as oil and natural gas, are non-renewable energy forms. How to maximize the oil or gas recovery is of great significance to the sustainable development of the global economy, and the enhanced oil recovery (EOR) technology has attracted worldwide attention, in which chemical enhanced oil recovery (CEOR) is one of the most effective ways. The construction of oil flooding systems based on new materials has always been the main direction of the development of chemical flooding for oil recovery. The application of nanoparticles in CEOR, which has developed rapidly in the past two decades, provides the basis for the application of graphene materials in CEOR [79,80,81,82]. The following studies show that the use of graphene and its derivatives is feasible in chemical enhanced oil recovery.

#### 3.2.1. Nanofluid Flooding

Nanofluid flooding technology shows great application prospects in CEOR. The term nanofluid refers to a homogeneous liquid obtained by highly dispersing nanomaterials (almost all of them are zero dimensional nanoparticles) in water or other liquid phases (such as ethanol). Compared with the conventional chemical flooding (alkali flooding, surfactant flooding, polymer flooding, etc.), the nanofluid flooding mechanism is more diversified, including conventional mechanisms such as mobility control, low interfacial tension, wetting reversal, as well as the unique interfacial effects of nanoparticles [83,84,85].

##### Graphene Oxide

**Figure 2:**
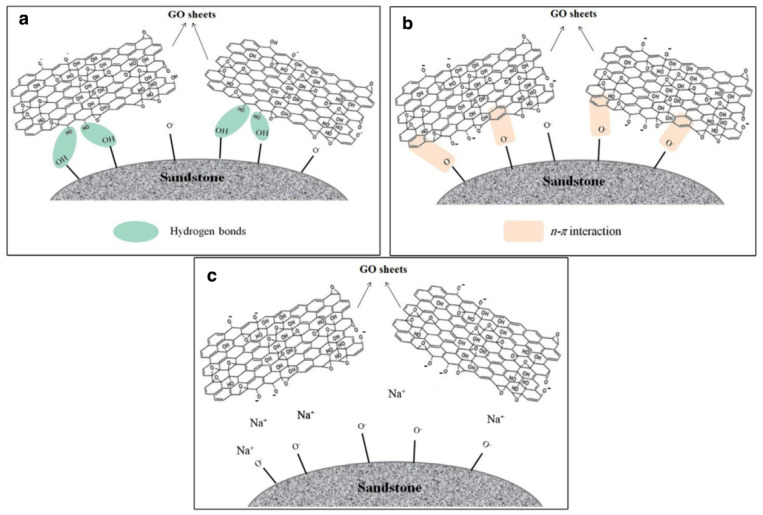
Schematic of adsorption mechanism of GO on sandstone at (**a**) low pH, (**b**) high pH and (**c**) at the presence of cations. (Reproduced with permission from [86]. Elsevier, 2017).

It is found that graphene oxide can be used in CEOR due to its surfactant-like properties, such as changing wettability, emulsifying property and reducing interfacial tension. Radnia et al. studied the adsorption behavior and mechanism of GO on the sandstone surface (as shown in Figure 2) and analyzed the change trends of adsorption under different conditions. They found that the adsorption amount of GO on the surface of sandstone was related to the concentration of GO solution. The higher the initial concentration was, the greater the adsorption capacity was. In addition, the adsorption amount can be increased by reducing the pH value and increasing the salt concentration. When the pH value was low, the hydrogen bond between the oxygen-containing functional groups on the GO sheet and the silicon hydroxyl group on the sandstone surface was the reason for the adsorption; when the pH value was high, the N-π interaction between -o-groups on the sandstone surface and π-electrons on the GO surface was the reason for the adsorption. Furthermore, the presence of the electrolyte increased the adsorption amount. It seems that Na^+^ cations acted as a bridge between the two negatively charged surface of the sandstone and GO sheets, and the bridging effect of Na^+^ was not as much as the effect of pH at low salinities [86].

**Figure 3:**
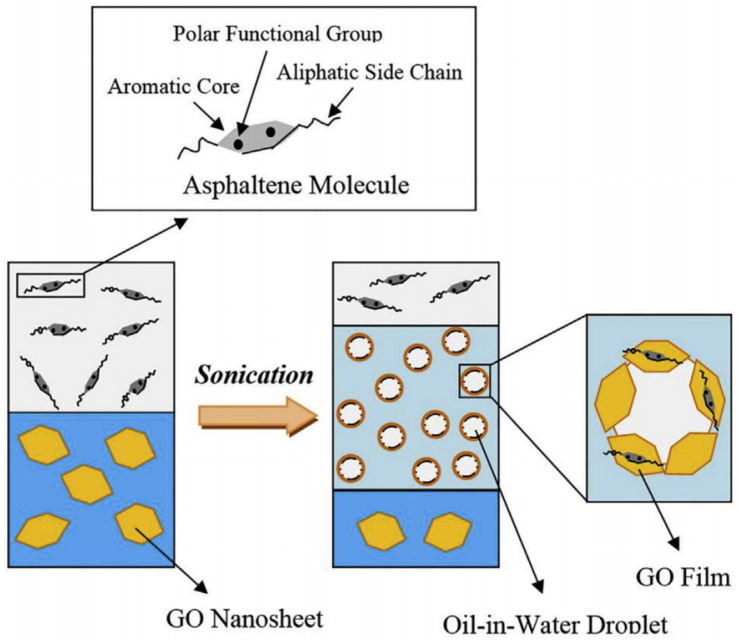
Schematic of emulsification process. (Reproduced with permission from [87]. Elsevier, 2019).

Then Radnia et al. studied the feasibility of GO in improving the oil recovery from the perspective of the emulsification performance of GO, as well as the effect of asphalt on the emulsification performance. The results showed that adding a small amount of asphalt in the oil phase can enhance the strength of the emulsion droplets film through the interaction between GO and asphalt, thus improving the emulsification efficiency and emulsion stability, as shown in Figure 3. In addition, the concentrations GO and asphalt had the most significant effect on the interfacial tension, which decreased with the increase of GO and asphalt concentrations. Furthermore, under all conditions, the type of emulsion was an oil-in-water emulsion, which was more suitable for enhanced oil recovery. However, water-in-oil emulsions were observed when the asphalt concentration was higher than 1.5 wt %. In order to control this phenomenon, the oil/water ratio should be increased [87].

Khoramian et al. studied the oil enhanced recovery capability of GO nanosheets from the perspective of their influence on the viscosity of water phase and emulsification performance. The results show that GO could increase the viscosity of aqueous solution, reduce the oil-water interfacial tension, and change the oil-wet carbonate rocks into water-wet. In addition, increasing the salt content in the aqueous solution could reduce the dispersion stability of GO on the one hand, increase the viscosity of the solution on the other hand, and meanwhile reduce the oil-water interfacial tension by a small margin. Furthermore, they proposed that the main mechanism of increasing recovery efficiency of GO was changing the mobility ratio. Therefore, through the above research, it is clear that GO can be used in chemical enhanced oil recovery [88].

##### Functionalized Modified Graphene

The functionalized modification products of graphene and GO can also be used in CEOR. The corresponding functional modifications mainly include alkylamine-modified graphene oxide, sulfonated graphene oxide, nitrogen-doped graphene, and graphene oxide/silica composite materials.

For alkylamine-modified functionalized GO, Luo et al. pointed out that the alkylamine-modified GO was structurally asymmetric amphiphilic Janus nanoparticles. They could automatically accumulate at the oil/water interface in brines with medium or high concentrations, and self-organize to reduce the oil/water interfacial tension. At the same time, this functionalized GO could form a layer of recoverable solid elastic film at the oil/water interface, separate the oil and water phase, and displace the oil phase. The oil recovery can be increased by 7.5% at an ultra-low dosage of 0.005 wt % and 15.2% at relatively low dosage of 0.01 wt %. Therefore, it can be used to improve crude oil recovery in secondary or tertiary oil recovery [89].

**Figure 4:**
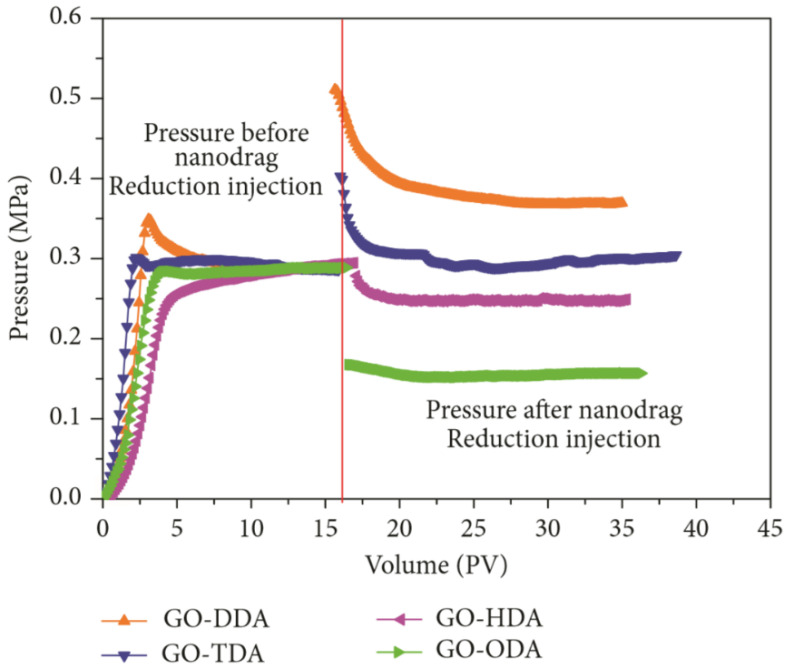
Pressure of water flooding before and after nano drag reducer injection [90].

Chen et al. found that increasing the length of the alkyl chain grafted on GO, increasing the surface roughness of the modified GO, and reducing the surface energy, could increase the contact angle of the surface of hydrophilic core. In addition, the hexadecylamine- and octadecylamine- modified GO have the ability to reduce the water injection pressure and increase the water injection capacity in low-permeability reservoirs (as shown in Figure 4), and can be used as nano-drag reduction agents in low-permeability reservoirs [90].

**Figure 5:**
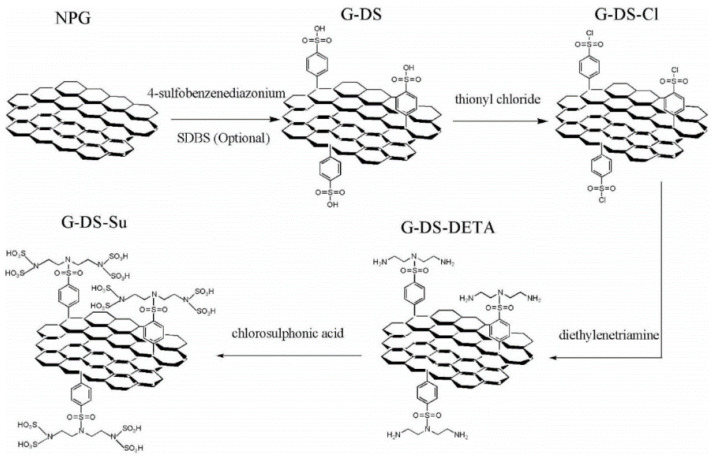
Schematic illustration for the preparation of functionalized NPG. (Reproduced with permission from [91]. Elsevier, 2018).

**Figure 6:**
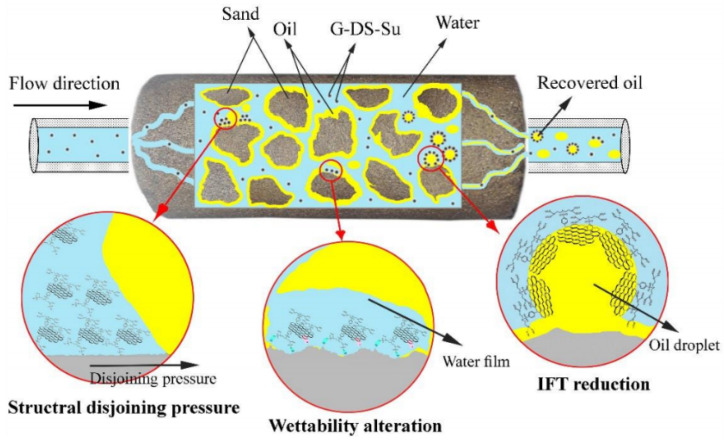
Schematic illustration for displacement mechanism of functionalized NPG. (Reproduced with permission from [91]. Elsevier, 2018).

Radnia et al. studied the ability and mechanism of sulfonated nano porous graphene (NPG) in improving oil recovery, as shown in Figure 5. The results showed that the sulfonated GO containing more sulfonated functional groups exhibited good dispersion stability in water. In addition, the sulfonated graphene could reduce the interfacial tension and change the wettability of the rock surface. At the same time, it was pointed out that the dominant mechanism of sulfonated graphene in improving oil recovery was its influence on rock wettability, as shown in Figure 6 [91].

Namin et al. pointed out that functionalized nitrogen-doped graphene could disperse stably in water in the condition of high salt and high temperature and could reduce the oil/water interfacial tension by 49.2%. Wettability reversal could be achieved by its adsorption on sandstone and limestone surfaces. In addition, when functionalized nitrogen-doped graphene was used as oil displacement agent, the oil recovery could be increased by 16.42%, which they believed was mainly due to its ability to reduce the interfacial tension [92].

AfzaliTabar et al. prepared rGO/SiO_2_ nanohybrid by the sol-gel method, and pointed out that the Pickering emulsion stabilized by this nano-porous graphene/SiO_2_ nanohybrid remained stable in a reservoir with a salt content of 1%, the temperature of 25–120 °C, and the pH value of 7–10. It is also pointed out that the composite material can reduce the oil/water interfacial tension, improve the rheology of the injecting fluid, and could be used in chemical flooding [93]. Tajik et al. also studied the use of silica-graphene nanohybrid in CEOR, but they focused on the effect of the number of oxygen-containing functional groups in the composites on CEOR. In addition, the influence of asphalt on its emulsification performance was also investigated, and the oil displacement performance was studied by using the micro flooding model [94].

#### 3.2.2. Polymer Flooding

Polymer flooding, which employs polymers as flooding agents, is a commonly used technique to improve oil recovery. The oil displacement mechanism is to reduce the water-oil mobility ratio and increase the sweep efficiency of the flooding fluid, thereby improving the crude oil recovery [95,96]. The common polymers used in this technology include natural polymers, such as xanthan gum, cellulose, etc., and synthetic polymers, such as partially hydrolyzed polyacrylamide (HPAM), polyacrylamide (PAM). Xanthan gum is not sensitive to salt, but its biological stability is poor, therefore, its application range is not as wide as synthetic polymer [97,98]. However, the rheological property of HPAM is susceptible to problems such as salt concentration, chemical degradation, and shear degradation, which limiting its use in high-temperature and high-salt reservoirs. In order to improve the rheological properties of HPAM in CEOR applications, the effects of different nanoparticles on their performance have been studied in recent years [99,100,101].

Haruna et al. prepared graphene oxide/partially hydrolyzed polyacrylamide materials (GO/HPAM) and studied the influence of GO nanosheets on the rheological properties and thermal stability of HPAM. The results show that hydrogen bonds were formed between GO and HPAM. The addition of GO significantly improved the long-term thermal stability of the material, as well as the storage modulus and loss modulus of HPAM. In addition, GO/HPAM composite has good performance under high salt conditions [102]. Lyu et al. prepared polyacrylamide/surface-modified graphene oxide composite (PAM/sGO) by using the copolymerization of sGO (made from that triethoxyvinylsilane covalently bonded to GO) and acrylamide (AM). The performance evaluation experiment should that the addition of graphene oxide could improve polymer’s temperature resistance, salt resistance and shear resistance, and could be applied to improve the oil recovery in high-temperature and high-salt reservoirs [103].

#### 3.2.3. CO_2_ Foam Flooding

In the process of CO_2_ foam flooding to improve oil recovery, in order to improve the foam stability, the stabilizer needs to be quickly migrated onto the surface of the liquid film to form a stable foam boundary, and slow down the drainage of the liquid film of the foam [104]. The commonly used foam stabilizers are polymers or surfactants. The mechanism of action involves stabilizing the foam by increasing the apparent viscosity of the liquid phase of the foam system, slowing down the speed of foam drainage and reducing the gas exchange rate between bubbles [105,106,107,108]. However, in high-temperature and high-salinity formations, the polymer would lose its stability due to its thermal decomposition, and the surfactant lacks long-term stability due to its poor adsorption capacity to the gas-liquid interface. In recent years, with the development of nanotechnology, scholars have studied the advantages of nanoparticle-stabilized foams, as well as the mechanism and influence factor, and have gained some recognitions. However, not many studies about the effect of graphene on foam properties and oil displacement effects were reported [109,110,111].

**Figure 7:**
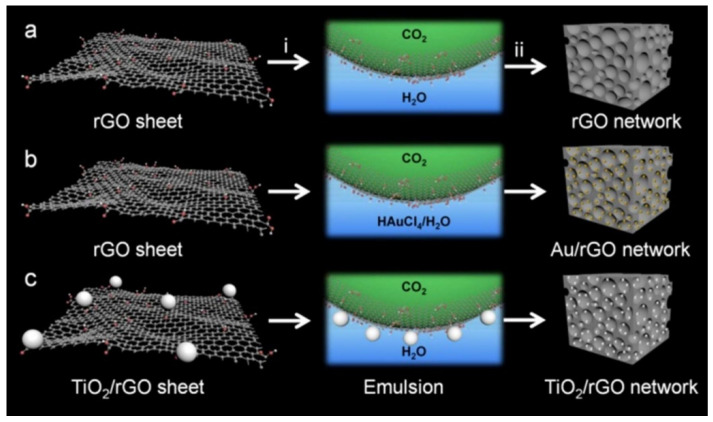
Schematic illustration for (**i**) forming emulsions in CO_2_ and water with the aid of rGO or rGO hybrid and (**ii**) constructing rGO network (**a**), Au/rGO network (**b**) and TiO_2_/rGO network (**c**) by freeze depressurization and drying for emulsions. (Reproduced with permission from [112]. ACS Publications, 2017).

Liu et al. demonstrated that partially reduced graphene oxide (rGO) can effectively stabilize CO_2_/water emulsion, as shown in Figure 7 [112]. Barrabino et al. studied the feasibility of graphene oxide, with the diameter of 4 μm–30 μm, nanometer GO (nGO, with the diameter of 70 nm–1.5 μm) and partially reduced GO (rGO, with the diameter of 260 nm–295 nm) as foam stabilizers in enhanced oil recovery by CO_2_ flooding. The results show that graphene oxide played a stabilizing role in the CO_2_/simulated seawater foam system due to the formation of hydrogels and graphene oxide accumulation effect. rGO could not stabilize the foam due to its small size, which increased the hydrophilicity. rGO with the content of 13–22% could not stabilize the foam, which was different from the results obtained by Liu. This may be due to the high reduction degree of the particles, and the increase of oxygen content would enhance the hydrophilicity and reduce the CO_2_-affinity [113].

### 3.3. Heavy Oil Recovery

Thermal oil recovery is the main method of heavy oil production, which refers to the method of injecting heat from the ground to the stratum or generating heat underground to achieve underground crude oil extraction [114,115,116]. At present, thermal methods, such as steam huff and puff, steam flooding, are mainly used for heavy crude oil production. On the one hand, the thermal recovery method can reduce the viscosity of heavy oil; on the other hand, it can break the carbon chain of the long carbon chain molecules in heavy oil and improve its fluidity [117,118]. Adding nanoparticles with high heat conductivity in the process of thermal recovery is helpful to improve the heat conduction efficiency and improve the viscosity reduction effect of heavy oil [119,120,121,122,123].

Elshawaf et al. studied the application of GO in heavy oil recovery. They pointed out that when the temperature was 40–70 °C and the GO dosage was 0.02–0.08 wt %, the viscosity of heavy oil can be significantly reduced by 25–60%. By comparing the viscosity reduction of heavy oil treated by other nano particles, such as Fe_2_O_3_, NiO, CuO, ZnO, Al_2_O_3_, TiO_2_ and WO_3_, Fe_2_O_3_ exhibited the best viscosity reduction effect. However, if the same heavy oil viscosity reduction rates were achieved, the price of GO was 10–15% lower than that of Fe_2_O_3_. Therefore, they claimed that GO not only had a significant viscosity reduction effect for heavy oil, but also was economically feasible, and can be applied to all methods of heavy oil production (such as steam huff and puff, steam flooding, SGAD, in-situ combustion, electromagnetic heating method, etc.) [124,125].

The use of microwave irradiation to extract heavy oil has the advantages of continuous heating, being unaffected by buried depth, easy to control, and a low-level of environmental pollution. At present, many countries and oil companies are carrying out relevant research and experiments. However, due to the small dielectric constant of heavy oil, the penetration depth of microwave in heavy oil reservoirs is limited, which affects the viscosity reduction effect [126,127,128,129]. Because of its high conductivity, thermal conductivity and aspect ratio, the two-dimensional sheet-like graphene could generate a strong electrical losses to microwaves. In addition, magnetic nanomaterials (such as Fe, Co, Ni and Co_3_O_4_) have strong magnetic loss to microwave. Therefore, by combining graphene with magnetic nanoparticles, graphene/magnetic nanocomposites with both electrical loss and magnetic loss can be obtained, which is conducive to widening the absorption band and impedance matching, and improving the microwave absorption capacity [130,131,132,133,134,135,136,137,138]. When applied to the development of heavy oil wells, the penetration depth of microwave can be increased, and the effect of viscosity reduction and stimulation can be improved.

**Figure 8:**
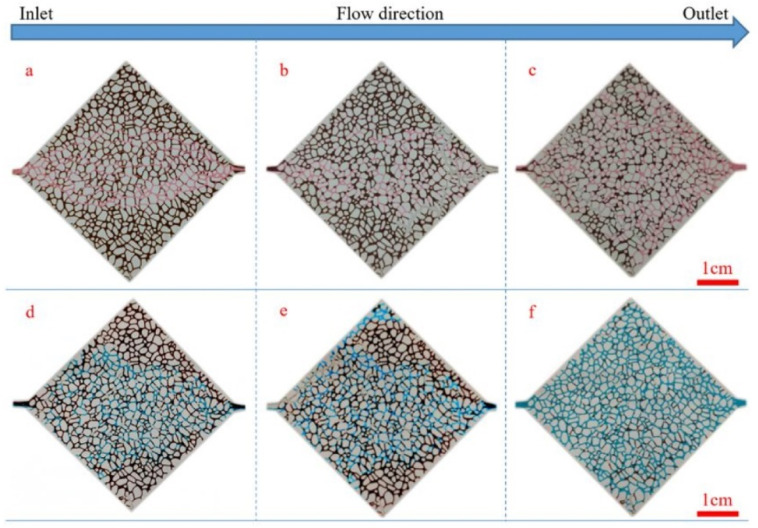
Comparison of the oil recovery effects between water flooding and magnetic graphene oxide fluid flooding in the microscopic model: (**a**) after water flooding, (**b**) microwave treatment for 5 min after water flooding, (**c**) subsequent displacement, (**d**) after MGO fluid flooding, (**e**) microwave treatment for 5 min after MGO fluid flooding, and (**f**) subsequent displacement. (Reproduced with permission from [139]. ACS Publications, 2019).

Xu et al. prepared magnetic graphene oxide (MGO) by a self-assembly method and conducted microwave-assisted enhanced recovery research. The results showed that MGO had good dispersibility in water. With the increase of microwave radiation time, the dispersibility weakened and formed magnetic reduced graphene oxide (MrGO), showing better lipophilicity and better microwave absorption ability when compared with MGO. MGO-assisted microwave irradiation showed good performance in reducing the viscosity of heavy oil. After microwave treatment for 10 min, the viscosity reduction rate reached 43.61%. In addition, the adsorption of MrGO onto the crude oil droplets could also reduce the viscosity. Compared with conventional water flooding, MGO assisted microwave flooding increased the recovery efficiency by increasing the sweep ratio, as shown in Figure 8. Therefore, MGO assisted microwave oil displacement technology was feasible in enhancing heavy oil recovery [139].

### 3.4. Profile Control and Water Shutoff

The heterogeneity of the formation makes the injected water directly flow into the oil well along a high permeable layer. The washing action of water to the high permeability layer further aggravates the heterogeneity, which makes it easier for water to break into the oil well along the high permeability layer [140,141,142]. In order to improve the sweep efficiency of the injected water, these high permeability layers must be blocked. Profile control and water shutoff is a common method for plugging the high permeability reservoir in oil fields. The water injection profile of the water injection interval can be adjusted by plugging the layer with high permeability from the water injection well, which is called profile control; the water production of the oil well can be reduced by plugging the water producer layer from the oil well, which is called water plugging of the oil well, or water shutoff [143,144].

Profile control and water shutoff are indispensable both in secondary oil recovery (i.e., water injection or gas injection) and tertiary oil recovery (special fluid injection). The mechanism of profile control and water shutoff to improve oil recovery is to improve the sweep efficiency [145,146,147]. In recent years, with the reservoir conditions that implementing profile control and water shutoff are more and more complex, the requirements for profile control agents and water shutoff agents also become higher. For this reason, scholars have conducted many studies, among which polymer nanocomposite gel system can be effectively used as profile control and water shutoff in the high-temperature and high-salt reservoirs [148,149,150].

Almoshin et al. prepared a kind of gel by using low molecular weight polyacrylamide (PAM, with the molecular weight of 550,000 Daltons), and ZrO_2_/rGO nanocomposite as the crosslinking agent. The novel polymer/graphene nanocomposite had a uniformly distributed three-dimensional network structure. The small size of the gel grid holes made the network structure thermally stable and firmly locked the water in the gel, even at high temperature. The improvement in the properties of this material was mainly due to the high specific surface area provided by graphene at the nanoscale, which increasing the interface interaction between the polymer and graphene. The experimental results of core flooding showed that the pressure drop of sandstone cores with high permeability (Berea sandstone with the permeability of 1000 × 10^−3^ μm^2^ and porosity of 21%) increased significantly after treatment. The material had good shear resistance and thermal stability. It can significantly enhance the plugging effect in high-temperature reservoir with lower cost, compared with the existing polymer gel [151].

**Figure 9:**
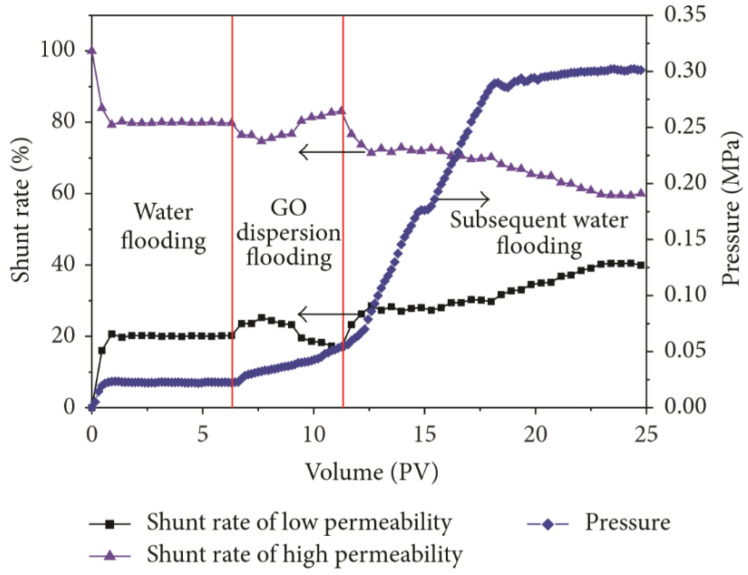
Pressure curves of the flooding experiment in the two parallel cores [152].

**Figure 10:**
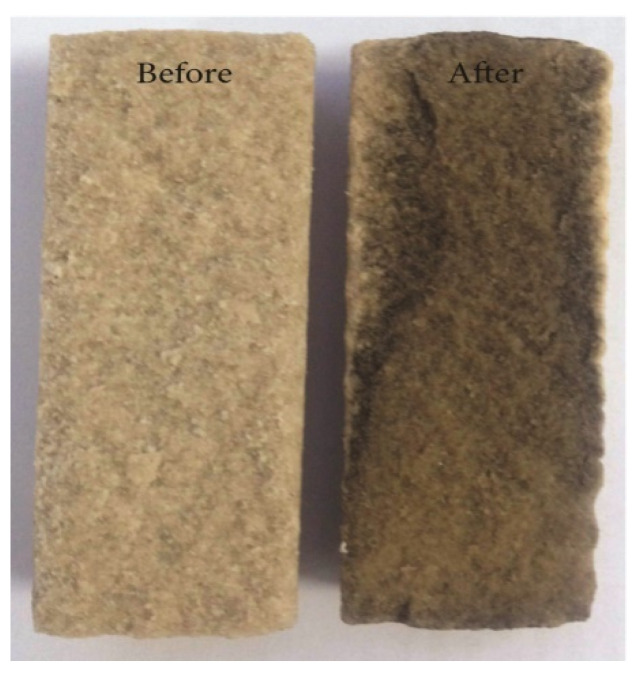
Image of the core profile before and after flooding [152].

Ye et al. found that GO had the function of adjusting the injection volume, plugging the high permeability layer and improving the sweep efficiency, making it can be used as profile control agent in water injection in the oilfield. Flooding experiments in the single core showed that the injection of GO dispersion into the core had two effects, one was to increase the injection pressure of the water phase, the other was to reduce the permeability due to the adsorption and retention of GO. As a result, the mobility of the displacement phase in the core was greatly reduced. Flooding experiments in the two parallel cores showed that in the stage of injecting GO dispersion, it preferentially entered the core with high permeability, and the adsorption of GO in the layer with high permeability caused physical plugging, which increased the injection pressure of high permeability core (as shown in Figure 9). In addition, due to the 3D pore structure of the GO particles dispersed in the pore structure, the plugging was not complete, and the pores in core still had a certain degree of connectivity (as shown in Figure 10) [152].

### 3.5. Tracer

Tracer technology is a very effective monitoring method to obtain valuable information about reservoirs in the oil and gas industry. As a material that can enter the reservoir with the fluid, tracers can judge the flow direction and seepage velocity of the dissolved fluid in the reservoir pores by tracking the movement path of the fluid, so as to understand the reservoir properties and reservoir conditions [153,154]. A good tracer should have the advantages of high detection sensitivity, good solubility with the injected fluid, small impact on the flow characteristics of the injected fluid, small adsorption capacity by the reservoir, strong anti-biodegradation ability, high temperature and high pressure stability, non-toxic or low toxicity, low cost and so on [155,156,157].

The commonly used oilfield tracers include chemical tracers, radioisotope tracers, and stable isotope tracers. Chemical tracers, including inorganic salts, fluorescent dyes, halogenated hydrocarbons and alcohols, are characterized by large consumption, high cost, poor adaptability and selectivity, low detection resolution, and environmental or personnel safety problems. Therefore, such kinds of tracers are gradually being eliminated. Radioisotope tracers are mainly some kind of tritium-containing compound, which has the advantages of small dosage, convenient detection and higher detection resolution. However, its radioactivity is harmful to the safety of personnel and environment, which limits its applications. The term stable isotope tracer refers to isotopes that are not radioactive, such as ^12^C, ^13^C, ^15^N, ^18^O, etc. There are few varieties of such tracers. Although there is no radioactivity in field application, they still need to be activated by an indoor atomic reactor after sampling, and their radioactivity can be measured only by atomic energy agency polarity indoor testing. Moreover, their analysis and test methods are complex and expensive. Therefore, these disadvantages limit their application. Carbon nanomaterials with adjustable fluorescence developed in recent years provide a new kind for tracers in oilfields [158,159,160,161,162,163,164].

Carbon quantum dots (CQDs) have significant quantum confinement effect and boundary effect due to their ultra-small size. They have adjustable band gap and can emit stable fluorescence when stimulated. They are new nano carbon materials with fluorescence properties, in which graphene quantum dots (GQDs) is a special CQDs [165]. Murugesan et al. studied CQD fluorescent tracers used for oil production and well detection. CQDs were prepared by electrochemical redox reactions. CQDs and nitrogen-doped CQDs showed stronger fluorescence signals and remained stable under conditions of 80 °C and API saline for 30 days. Core flow experiments showed that the recovery of CQDs in 1 wt % NaCl solution was 76% in cores with low permeability. CQDs thus provide a new alternative for tracers for monitoring reservoir conductions [166].

### 3.6. Oily Wastewater Treatment

Oily wastewater in oilfields refers to all kinds of sewage generated during the process of drilling, production, and transportation, mainly including drilling sewage, wastewater separated from crude oil and other types of oily wastewater [167,168,169]. Oily wastewater has the characteristics of large amount, complex composition, difficult degradation and wide pollution range. In particular, the large-scale application of tertiary oil recovery technology in oilfields produced a large volume of wastewater with high emulsification, high viscosity and high oil content, which increases the difficulty of oilfield wastewater treatment [170,171,172]. In addition to petroleum hydrocarbons, oily wastewater also contains sulfur, phenol, cyanogen and other harmful substances. If it is discharged into the environment without treatment, it will destroy the ecological balance of receiving water bodies, cause great harm to the natural environment and human health, and cause huge losses to the production and life of human society. Therefore, it must be effectively treated to meet the standards before it can be discharged [173,174,175].

Materials with adsorption and separation abilities are often used in oily wastewater treatment [176,177,178]. Three-dimensional graphene, which is composed of two-dimensional graphene as the basic unit, has the characteristics of rich pores, high surface area, and the hydrophobic and lipophilic properties. It shows unique advantages in the adsorption and separation, and gradually becomes a new functional material for adsorption and separation. In addition, the hydrophobic and lipophilic properties of the graphene surface can be used to construct a surface adsorption layer for oil-water separation. In recent years, graphene-based sponges, graphene gels, graphene/carbon nanotube composites and graphene-based separation membranes have been reported more and more in the field of adsorbent and oil-water separation. The above materials not only have a large oil absorption capacity, but also exhibit excellent recycling characteristics [179,180,181,182,183].

Nguyen et al. prepared graphene nanosheets-based sponges with high oil absorption, good selectivity and recyclability by a dip coating method. The material had super-hydrophobicity and super-lipophilic characteristics, and the adsorption capacity was up to 165 times of its own weight. It was suitable for oil and organic solvent absorption [184]. Also, Qiu et al. prepared super-hydrophobic reduced graphene oxide/melamine sponge (rGO-MS) by modifying the surface of melamine sponge (MS) with GO. The material had excellent adsorption capacity for both the oil floating on water and the heavy oil under water, and still maintained more than 90% adsorption capacity after 50 times of adsorption-extrusion tests. The separation efficiency of oil-water mixture under static and agitating conditions was as high as 4.5 × 10^6^ and 3 × 10^6^ L/(m^3^·h), respectively [185].

By adjusting the surface roughness and surface energy, Yang et al. designed a graphene foam material with super hydrophobic characteristics, which had good adsorption properties for both oil and a variety of organic solvents [186]. The enhanced PVDF/graphene composite oil-absorbing film (NR-PGM) of PET-PA non-woven cloth prepared by Zhang tai et al. had super-hydrophobicity and super-lipophilic characteristics, and the separation efficiency was up to 97.0%. In addition, the separation efficiency remained above 95.0% after 10 times of repeated tests [187]. Graphene foam with the wettability switchable characteristics response to pH value was prepared by Zhu et al. It can adsorb 196 times of its own weight and can be recycled used for more than 10 times [188,189]. Naseem et al. prepared TiO_2_/GO/rTAc membrane by introducing GO and TiO_2_ coating into cellulose triacetate (TAC) membrane by electrophoretic deposition. The oil-water separation rate could reach up to 98.9%, exhibiting broad application prospect in oil-water separation [190]. Hu et al. prepared a GO modified Al_2_O_3_ membrane using GO as the modifier by vacuum transfer method, which has longer service life and better separation performance compared with the unmodified membrane [191].

### 3.7. Corrosion Prevention Coating

In the process of oil-gas storage and transportation, crude oil, refined oil and natural gas are all corrosive to equipment and pipelines. With the development of reservoirs with harsh conditions such as high temperature and high salinity, higher requirements are put forward for related metal materials and rubber materials, such as abrasion resistance, corrosion resistance and higher strength [192,193].

The application of nanotechnology in oil and gas equipment and pipelines is attracting increasing attention in the field of oil and gas industry, especially the application research of nanocomposites. The discovery of carbon nanotubes and graphene with higher strength makes the application of nanocomposites in the field of oil and gas industry become a new hotspot. Nanocomposites refer to the complex system that was prepared based on resin, rubber, ceramic and metal as the continuous phase, and metal, semiconductor, rigid particles and other inorganic particles, fibers, carbon nanotubes and other modifiers as the dispersion phase. The modifier is evenly dispersed in the matrix material by an appropriate preparation method. The equipment and apparatus prepared based on nanocomposite materials have the characteristics of light weight, corrosion resistance, fire resistance, durability and high strength. Nanocomposites can replace some of the metal material components in the oil and gas industry (such as tools and accessories in offshore oil platforms, transportation vehicles, and drilling), or as sealing materials (such as packers and sealing ring), or as coating agent or lubricants [194,195,196,197,198].

Graphene has been widely used in anticorrosive coatings due to its advantages of large specific surface area, good toughness and good self-lubricating property. The graphene-modified coating has a tight structure with improved corrosion resistance and wear resistance. Graphene-modified anticorrosive coatings are a good choice for oil pipe protection [199,200,201]. Halliburton’s downhole seals (packers, sealing rings) made of graphene and a variety of other nanocomposites, can deal with explosive decompression and sealing failure, such as expansion, thread damage, wear and thermal degradation. In addition, the coating of graphene and its derivatives on the surface of oil and gas equipment and pipelines can improve the strength and corrosion resistance [202]. The results of the study conducted by Singhbabu et al. showed that in high salt environment, compared with uncoated cold rolled steel, the corrosion rate of GO coated steel was reduced by more than 10,000 times [203]. Cao et al. studied the characterization method of graphene-modified coatings for oil pipes. They also pointed out that, due to the large specific surface area of graphene, adding a small amount of graphene can significantly improve the performance of coatings. Compared with other advanced anticorrosive coatings, graphene-modified anticorrosive coatings were lower in price, exhibiting a broader application prospect [204].

### 3.8. Tools and Apparatus

#### 3.8.1. Tools

During the drilling process, drill bit and downhole power apparatus are the main tools used to break rocks. The harsh environment of high temperature and high pressure put forward higher requirements for drilling tools. Graphene coatings have excellent mechanical properties, which can optimize the surface morphology and characteristics of metal, improve the wear resistance, corrosion resistance and impact resistance of drilling tools, and prevent the tool surface from oxidation and rusting. Chakraborty et al. coated the surface of diamond particles with a graphene film and applied it to PDC (polycrystalline diamond compact) drilling bit, which extended the drilling life, and meanwhile the temperature resistance reached 1200 °C [205]. Keshavan et al. also added graphene to the diamond particle material of PDC bits to improve the wear resistance, thermal stability and impact resistance of the bits [206].

Rubber failure is one of the common failures of downhole tools in petroleum engineering. Ocsial company added graphene nanotubes concentrated solution with a mass fraction of 1.7% to nitrile rubber, which increased the tensile modulus of nitrile rubber by about 30%. When the modified nitrile rubber was applied to the rubber stator used in the screw drilling tools, the wear resistance of rubber stator was increased by 20% and the mechanical drilling speed was also increased by more than 20% [207].

#### 3.8.2. Sensors

Sensors are crucial for finding new oilfields in the process of exploration, safety monitoring in the process of stimulation treatments and data collecting in oil and gas production [208]. In oil and gas exploration, optical waveguides are often used to detect the geological characteristics of the target layer, downhole environmental parameters (including temperature, pressure, etc.) and the characteristics of fluids near the well. Waveguides include optical fiber cables, optical fiber sensors and other optical components. In the hydrogen-rich environment of the wellbore, the free hydrogen atoms diffuse into the waveguide and react with the defective sites in the optical fiber, which affects the transmission of light in the waveguide and leads to a weak signal quality, which is called “hydrogen dimming” [209,210,211]. Bhongale et al. proposed attaching graphene to the waveguide surface as a protective layer. The excellent mechanical properties of graphene can extend the service life of the waveguide, prevent the diffusion of hydrogen atoms, and weaken the “hydrogen darkening” phenomenon. In addition, graphene has high light transmittance, which can improve the clearness of signal as a protective layer [212].

In order to ensure the quality of spatial resolution, it is often necessary to use multiple acoustic sensors at the same time in oil exploration. The development of acoustic sensors is directly affected by the development of lightweight diaphragm materials [213,214]. At present, the conventional sensors based on silicon or silicon dioxide materials have the problem of low sensitivity. In addition, although the sensors based on polymer materials have high sensitivity, their mechanical strength is limited, and they are unstable in the condition of pervasion structure and water vapor existed environment [215]. Ma et al. developed an F-P pressure sensor with a pressure sensitivity of 39.2 nm/kPa by using a 25 μm ϕ graphene diaphragm. After that, an optical fiber F-P acoustic sensor was developed by using a graphene film with the thickness of about 100 nm and the diameter of 125 μM. Its dynamic pressure sensitivity was as high as 100 nm/kPa, and it can detect a minimum acoustic pressure signal of 60 μPA/Hz^1/2^ [216,217].

The poor conductivity of oil-based drilling fluids could block the direct current, resulting in the unavailability of resistivity logging while drilling. Magnetic graphene nanoribbon stacks (MGNRs) are quasi-one-dimensional graphene-based materials. Their special edge confinement effect makes their properties flexible and adjustable. For example, the magnetic graphene material cannot be used directly because there is no gap between the conduction band and valence band, but it can be applied to the field effect transistor (FET) when it is cut into a smaller scale MGNRs [218,219]. Genorio et al. attached MGNRs on the surface of oil-based drilling fluid particles as a conductive coating to improve the reliability of information transmitted by sensors in the wellbore. In addition, because of the size of the MGNRs can reach nanometer level, so it can enter into pores and fractures with smaller pore sizes to detect the location of the remaining oil and gas, especially for shale reservoirs which are rich in nanopores and microfractures [220].

Graphene, as a functional reinforcement material, can enhance the conductivity of composite materials. Compared with other conductive materials, graphene has excellent conductivity, certain degree of flexibility, and good compatibility with organic polymers. It is an ideal material for the preparation of flexible conductive composite materials with organic polymer. Graphene flexible sensors enhance the selectivity of commonly used sensors in oil and gas exploration [221,222,223]. The graphene-based fiber temperature sensor developed by some Chinese scholars had the advantages of quick response speed (greater than 0.0228 °C/s), high sensitivity (0.134 dB/°C), high accuracy (0.03 °C), long service life, strong anti-electromagnetic interference ability and strong repeatability, which could be used in fluid media [224]. Tian et al. used graphene as the resistive material and made use of the extrusion film effect to develop a pressure sensor with a sensitivity as high as 0.96 kPa^−1^ (45 times higher than that of the silicon piezoresistive pressure sensor) in a wide pressure range (0–50 kPa). The response time under high pressure reached 0.4 ms, which can be promoted in the field of oil and gas exploration [225].

There are many types of pipelines used in oil and gas production, and the performance requirements for pipelines are various according to the different construction situations. When the oil field enters the middle or high water cut stage, the properties of produced fluid in oil well has changed, and the partial pressure of the corrosive gas such as CO_2_ and H_2_S in natural gas well will gradually exceed the critical value. The corrosion medium will lead to fracture and dropout of rod or tube phenomena, and even cause the scrapping of oil and gas wells. Therefore, the detection of CO_2_, H_2_S and other gases is worthy of attention. The graphene-based gas sensor had ultra-high sensitivity and ultra-low detection limit because every atom was able to interact with gas molecule, providing a new choice for oil field gas sensor [226,227,228,229].

In the medical field, bones and tissue structures can be distinguished by contrast agents. Similarly, in the field of oil and gas field exploration and development, the fluids and reservoir rock skeletons can also be distinguished by contrast agents, so that the direction of fluid flows can be analyzed and the three-dimensional fracture morphology of the reservoir can be identified. The research of contrast agents based on graphene and its derivatives is one of the hotspots in the biomedical field. Many scholars have shown that the contrast agents based on graphene and its derivatives has good stability, high relaxation rate and easy to monitor and trace. Therefore, contrast agents based on graphene and its derivatives can also be extended to the field of oil and gas industry, expanding their application scope [230,231,232].

## 4. Conclusions

As new materials, the development of graphene and its derivatives needs to go through a gradual process from laboratory discovery to large-scale application, and this should follow the development rules of emerging industries. The application of graphene and its derivatives in the upstream oil and gas industry is still in the initial exploring stage, but with its excellent mechanical, chemical, electrical and optical properties, graphene and its derivatives have broad application prospects in hydrocarbon exploration, oil and water well stimulation, downhole operation, enhanced oil recovery, oilfield wastewater treatment, tools and apparatus, and other aspects. Limited by the cost and the depth of the research, there is still a certain gap between the applications of graphene in the oil and gas industry.

In order to promote the application of graphene and its derivatives in the oil and gas industry, further research should be conducted in the following aspects: (1) clarification of the microscopic mechanisms, such as the role of graphene materials in chemical enhanced oil recovery; (2) broadening the research scope in the oil and gas industry, such as the application of graphene materials in shale gas production; (3) revealing the compatibility with other additives, such as the compatibility of graphene materials with surfactant in chemical flooding; (4) putting forward performance requirements for graphene and its derivatives used in the oil and gas industry, such as functional modification of graphene for different purposes in profile control and water shutoff treatments. In addition, how to reduce the cost of industrial application of graphene in the oil and gas industry is particularly important given the current low oil price situation.
